# Visualising Primary Health Care: World Health Organization Representations of Community Health Workers, 1970–89

**DOI:** 10.1017/mdh.2018.40

**Published:** 2018-10

**Authors:** Alexander Medcalf, João Nunes

**Affiliations:** 1 Centre for Global Health Histories, Department of History, Berrick Saul Building BS/120, University of York, Heslington, York YO10 5DD, UK; 2 Department of Politics, University of York, Heslington, York YO10 5DD, UK

**Keywords:** Primary health care, Community health workers, World Health Organization, Public information, Global health, Photography

## Abstract

For the World Health Organization (WHO), the 1978 Alma-Ata Declaration marked a move away from the disease-specific and technologically-focused programmes of the 1950s and 1960s towards a reimagined strategy to provide ‘Health for All by the Year 2000’. This new approach was centred on primary health care, a vision based on acceptable methods and appropriate technologies, devised in collaboration with communities and dependent on their full participation. Since 1948, the WHO had used mass communications strategies to publicise its initiatives and shape public attitudes, and the policy shift in the 1970s required a new visual strategy. In this context, community health workers (CHWs) played a central role as key visual identifiers of Health for All. This article examines a period of picturing and public information work on the part of the WHO regarding CHWs. It sets out to understand how the visual politics of the WHO changed to accommodate PHC as a new priority programme from the 1970s onwards. The argument tracks attempts to define CHWs and examines the techniques employed by the WHO during the 1970s and early 1980s to promote the concept to different audiences around the world. It then moves to explore how the process was evaluated, as well as the difficulties in procuring fresh imagery. Finally, the article traces these representations through the 1980s, when community approaches came under sustained pressure from external and internal factors and imagery took on the supplementary role of defending the concept.

## 1 Introduction

The discussions surrounding the 1978 International Conference on Primary Health Care (held in Alma-Ata, Kazakhstan) and the ensuing Alma-Ata Declaration marked a fundamental shift in the ideational makeup of the World Health Organization (WHO). Indicating a move away from the disease-specific and technologically-focused programmes of the 1950s and 1960s, the Declaration provided a reimagined strategy to provide ‘Health for All by the Year 2000’. This approach was centred on the concept of primary health care (PHC), which encompassed an ambitious vision to provide essential health care that was based on acceptable methods and appropriate technologies, devised in collaboration with communities and dependent on their full participation. Representing a break with the strategies of previous decades, the WHO recognised the need to convince multiple audiences of the efficacy of this shift. This was pursued by outlining a hopeful vision of the future, by showcasing examples where PHC was already underway, and by publicising appreciable results.

This article examines the complex processes through which the WHO sought to present and ‘sell’ PHC to governments, donors and populations around the world. The conviction that the use of (Western) technologies was the best strategy for solving the world’s health problems had been ardently advocated for decades, including by sections of the WHO. As the agency shifted gears in the run-up to Alma-Ata it attempted to develop attractive means for communicating the approach to different audiences at a time when many (and not least among WHO staff) were unsure about it. The WHO dedicated significant resources to producing information materials in order to convince those who were unenthusiastic or sceptical. However, the challenges associated with this task increased in the 1980s when, despite well-founded ideas and impressive rhetoric, the approach proved far from a panacea for deep-set problems. There quickly arose a disjuncture between aspirations and results, which only widened in the ensuing global economic recession.

Considering this process is important because it sheds new light on the WHO’s approach to PHC, and how it attempted to unify disparate groups in the face of criticism. As Margaret Jones and Chandani Liyanage have recently argued, PHC has been subject to shifting and at times competing interpretations.^1^ Whereas scholars have explored the origins of PHC, and the challenges of this change in direction within the WHO,^2^ this article examines the WHO’s efforts to promote PHC chiefly through visual representations, mainly in the agency’s public-facing magazine *World Health*, scrutinising how programmes were depicted in an increasingly crowded ‘marketplace’ of images and ideas. Scholars working across disciplines have examined how health issues have been presented, and how narratives about disease, suffering and rescue were constructed both textually and pictorially.^3^ They have demonstrated the important role of imagery in not just picturing health issues but shaping mindsets and attitudes. The value of visual media to spread hopeful messages and counter harmful ones had been a central component in the work of the United Nation’s (UN’s) specialised agencies since the late 1940s.^4^ Yet, in the specific case of the WHO, visual media has only recently attracted scholarly attention.^5^ The WHO’s own dedicated Division of Public Information, established in 1948, coordinated press releases, liaised with media officials and oversaw the production and organisation of films, exhibitions and photo stories.^6^ Photography in particular was used for communicating the WHO’s work and mission to the general public, policy-makers and decision-takers through exhibitions and magazine photo stories which were developed in collaboration with leading photographic agencies such as Magnum.^7^

However, the fact that the WHO had used mass communications strategies to publicise its main initiatives, and had in the process become adept at spreading certain messages far and wide, meant that the efforts to promote PHC needed to displace some very well-established notions and dominant visual narratives. One of the most vigorously propounded was the focus on ‘miraculous’ medical interventions and ground-breaking technologies in the hospital environment. As David and Rodogno demonstrate, in general, and for many years, a chief visual device used by the WHO was the ‘before and after’ narrative, an attempt to unequivocally demonstrate the remarkable benefits of the drugs given to people suffering from conditions such as yaws, malaria and leprosy.^8^ Similarly, health professionals including doctors, nurses and laboratory technicians were commonly presented as heroes, saviours or inspirational individuals who held the keys to better health. The WHO therefore contributed to a particular vision about health advancement aligned to technological progress.^9^

PHC required a different focus, and, in the WHO’s new visual strategy, community health workers (CHWs) played a central role as key visual identifiers of the push towards Health for All. Nowadays, CHW is the name commonly given to close-to-community workers with no specialised medical training who traditionally operate as links between doctors, nurses and remote or hard to reach groups. They can specialise in one task or carry out a diversity of functions, which commonly involve identifying the health needs of local populations, particularly of neglected groups like women, the elderly and the disabled; gathering epidemiological information; scheduling consultations; accompanying patients in long-term medication protocols; supporting vaccination programmes and vector-control interventions; and promoting health education and disease prevention campaigns. In some shape or form, people had undertaken these roles for several decades, albeit under different names. The term CHW was a relatively late attempt to bring some unity. Historians such as Marcos Cueto and Randall Packard have reconstructed some aspects of the complex history of these workers while examining the push for PHC.^10^ Famous examples hailing from individual countries, such as China’s ‘barefoot doctors’, have also attracted some consideration.^11^ From the 1970s onwards, the WHO assumed a central role in championing CHWs. Believing that for many developing countries CHWs were the most realistic solution to provide essential health coverage to the total population,^12^ the WHO used the Alma-Ata conference to energise the CHW model. The ultimate objective was to heighten public and political interest in giving power and responsibility for health back to communities, raise the visibility of PHC, and overturn negative or ambivalent attitudes towards it.

This article examines a period of intense picturing and public information work regarding CHWs, centred around the 1978 Alma-Ata Declaration and the ensuing championing of PHC. It tracks attempts to define CHWs and examines the techniques employed by the WHO during the 1970s and early 1980s to promote the concept to different audiences around the world. In this sense, the article breaks new ground in understanding how the visual politics of the WHO changed to accommodate PHC as a new priority programme from the 1970s onwards.^13^ It then moves to explore how the process was evaluated and the difficulties in spreading the message and procuring fresh imagery. Finally, the argument traces these representations through the 1980s and early 1990s, when community approaches came under sustained pressure from external and internal factors and imagery took on the supplementary role of defending the concept.

## 2 PHC and the Community Focus

The WHO Constitution, which came into force on 7 April 1948, defined health as ‘a state of complete physical, mental, and social well-being and not merely the absence of disease or infirmity’,^14^ and, in pursuit of this, the agency was initially created with a very broad remit which included: planning, coordinating and evaluating activities concerned with strengthening public health and hospital administration; mental and dental care; environmental sanitation; nursing services; health education of the public; as well as promoting maternal and child health, nutrition and occupational health.^15^ It was nevertheless acknowledged that establishing adequate provisions for such an all-encompassing understanding of health would take years to bring about. At a time of heightened need to demonstrate the value of WHO’s existence, and one characterised by the increasing availability of technological interventions, a parallel interest in mass campaigns focused on specific diseases took hold. Although some of the chief architects of the WHO had been proponents of social medicine, the agency’s early years were characterised by a widespread interest in vertically integrated programmes against diseases such as yaws, malaria and tuberculosis, part of what has been described as a ‘narrowing vision’.^16^

The post-war faith in ‘miraculous’ interventions based on technology and pharmacological innovations aligned with the perception that human ingenuity, and particularly scientific knowledge, would deliver solutions to rid humans from the scourge of ancient maladies. At the WHO, a culture of competition arose between the disease control programmes on the one hand, and the need to strengthen basic health services on the other. In addition to pursuing different objectives, each of these strategic directions relied upon different forms of funding, types of professionals and power structures.^17^ Overall, however, as the world turned to ‘magic bullets’ in the form of vaccines, antibiotics or chemical-based interventions (such as DDT), interest in community health programmes waned.

Yet, by no means did it disappear entirely. By the late-1960s the disease-focused programmes were faltering, with significant results harder to come by than originally projected. As it became evident that the envisioned success of these strategies would not be realised, thoughts returned to strengthening basic health services, and balancing vertical eradication programmes with ‘horizontal’ approaches aimed at improving health systems.^18^ For instance, in the foreword to the agency’s annual report for 1966, the WHO Director-General Dr Marcolino Candau (the WHO’s second Director-General, appointed in 1953) stated that the success of practically all the agency’s activities depended upon establishing and strengthening basic national health services.^19^ A 1972 WHO working group on research into the organisation of community health services concluded that attempts to transpose organisational patterns from developed to less developed countries had led to ‘unsatisfactory outcomes’ or had ‘simply failed’.^20^ This, along with several other studies and reports, recommended further avenues for research in the field of the organisation of community health.

This aligned with a more general vogue for ‘thinking small’ in the late 1960s and early 1970s, advocated in books such as E. F. Schumacher’s *Small is Beautiful: A Study of Economics as if People Mattered* (1973), which stressed the need for community development and community-centred approaches rather than top-down programmes.^21^ The historian Walter Bruchhausen also describes how Christian international health organisations came to the conclusion that the starting point for improving health should be communities, ‘yet the word “community” could be ambiguous and was as often used to name the target rather than the agent of health care’.^22^ The Christian Medical Commission’s publication, *Contact* magazine, which first appeared in 1971, was aimed at ‘those searching for new forms of health more relevant to existing needs than the traditional system’.^23^ As such, early issues gave prominence to topics such as community medicine, community health projects and village health workers, providing detailed reports and case studies. Ideological shifts were also buttressed by a changing operational context. In particular, the burgeoning influence of the New International Economic Order (NIEO), a movement which sought to address the imbalance of power and influence between developing and developed countries, brought to the fore questions of development and social justice, and provided the background for a greater emphasis on national healthcare capacities.^24^

At the WHO, Candau’s successor as Director-General in 1973, Dr Halfdan Mahler, was a supporter of the community approach, and in order to raise its profile he encouraged staff to read *Contact*.^25^ Mahler became known for his zeal in advocating community.^26^ At a meeting of the Regional Advisers of Community Health Services in 1974, he insisted that the WHO should do more in order to promote self-reliance and self-sufficiency. He condemned the WHO’s inability to learn from failures, and insisted that it should assist and participate, but that it had ‘no right to impose on Governments’. Mahler reiterated the need for ideas which did not necessitate highly sophisticated methods or tremendous resources.^27^ To investigate the potential of such initiatives, the WHO sponsored a study titled *Health by the People*, edited by Kenneth Newell, Director of the WHO division Strengthening of Health Services. The study was published in 1975 and comprised ten country-focused case studies. The WHO’s motives, as described in the introduction, were to identify the problems that the world faced and ‘present successful solutions to them, in the hope that information about existing successes will encourage others to seek out new paths’.^28^ It broadly argued that preventive and curative health care at the community level were often achieved in situations where the population took responsibility for their health in collaboration with health services, and that mass mobilisation of people made previously untapped resources available to support health-related activities. Newell noted a number of consistencies between the studies within, not least the ‘formation, reinforcement, or recognition of a local community organization’.^29^

In 1975, a joint UNICEF/WHO study on alternative approaches to meeting basic health needs in developing countries restated that conventional health services, organised along Western lines, were unlikely to expand to meet the basic health needs of all people, and that the time had come to ‘take a fresh look at the world’s priority health problems and at alternative approaches to their solution’.^30^ This report recommended that the agencies ‘should adopt an action programme aimed at extending primary health care to populations in developing countries, particularly to those which are now inadequately provided with such care, such as rural and remote populations, slum dwellers and nomads’.^31^ As this process of redefining problems and solutions took hold, communities increasingly took centre-stage. This was not limited to Geneva; O. Adeniyi-Jones, the Director of Health Services of the WHO Regional Office for Africa, recommended that maintaining health required ‘active and continuing participation of the individual and the community to which he belongs’. Community involvement, in his view, meant not just public participation in campaigns, but also ‘sharing the responsibility and participating actively in planning and organizing health services’.^32^ Around the same time, reports about sanitation programmes in the African region extolled the importance of community action and of ‘harnessing the community spirit…in the battle against unhygienic conditions’.^33^

This appetite for community approaches turned into a fundamental rethinking of health provision in the form of PHC. The Alma-Ata conference was intended to promote PHC to all member countries, exchange experiences and information on PHC, and define its principles and the operational means of overcoming practical problems.^34^ The conference was envisioned as a unique opportunity to heighten public and political interest in giving power and responsibility for health back to communities. Community health workers, as will be explained in more depth in the next section, were hailed as the most realistic solution to provide essential health coverage to the total population for many developing countries.^35^ It was recognised that ‘the most serious health needs cannot be met by teams with spray guns and vaccinating syringes’, but must be tackled from within the communities themselves.^36^ The accompanying Alma-Ata Declaration emphasised that the people should have the right and duty to participate individually and collectively in the planning and implementation of their health care, and that PHC, defined as ‘essential health care based on practical, scientifically sound and socially acceptable methods and technology made universally accessible to individuals and families in the community through their full participation and at a cost that the community and country can afford to maintain…’, was the key to achieving the target of health for all by the year 2000.^37^

## 3 ‘A Different Type of Drama’: Alma-Ata and the Rise of CHWs

The supporters of PHC at the WHO recognised the pressing need to increase the former’s visibility and credibility with other international agencies, with the public, and even among the agency’s own staff. As historian and retired WHO Senior Scientist Socrates Litsios recalls, there was a ‘mixed reaction’ to this new priority within the organisation.^38^ Messages which ran counter to, or suggested that Western approaches were no longer appropriate for all, and even held back development in cases, were distrusted in certain quarters. Referenced by the WHO’s Strengthening Health Services officials, the model of Western medicine was one ‘still copied, sometimes slavishly, in many developing countries to their detriment…all too often an innovative programme in a developing country is seen by many as something second-rate because it does not follow the established system’.^39^ The widely-sponsored narrative about technologically-driven progress, and a commensurate heroic vision of Western medical professionals, was referenced in the background documents to Alma-Ata, which reported that the ‘position, importance and status of the physician in Europe goes a long way to explaining why “health by the people” is almost universally rejected in the European Region’.^40^ Similarly prevalent was the corresponding assumption that people in developing countries were incapable of taking health matters into their own hands, in part because of ‘backward’ traditions that stood in the way of effective health advancement. Even the WHO’s magazine, *World Health*, had included stories in the 1950s and 1960s which pictured modernity ‘coming to town’ to protect needy populations.^41^

Therefore, a crucial strategy to garner support was to promote model examples of PHC through the WHO’s media outlets. At the crux of this lay the need to communicate an outwardly simple idea, summarised in the 1978 report on PHC: to ‘make people appreciate that primary health care is realistic, since it provides, at a cost that can be afforded, essential health care for all in a spirit of social justice rather than sophisticated medical care for the few…’.^42^ Despite the growing importance of television as a communications medium, throughout the 1970s photographic journalism continued to be an effective means of introducing the general public to the health situation around the world. *World Health*, the WHO’s premier public-facing photo magazine, was visually lavish; hard-hitting text was accompanied by powerful splash-page photographs which were frequently presented as a way to see the world through the ‘eyes’ of the WHO. In 1973 *World Health* circulated around 220 000 copies, with issues appearing in English, French, Portuguese, Russian and Spanish. The stories often reached further afield, as their reproduction in leading popular publications was actively encouraged.^43^ This was an expeditious way of putting the WHO’s work and viewpoint in front of a greater worldwide audience: more than 40 000 photographs were requested in 1975 alone.^44^ Given the importance of the medium for communicating the WHO’s mission and programmes, the years leading up to and immediately after Alma-Ata represented an intense period of photographing and visually representing PHC, with twenty-five photo missions made to nineteen different countries. The WHO featured the fruits of these assignments in special issues of *World Health* (whereby the entire number was given over to PHC), reprinting dozens more in stand-alone articles and making the content available to editors and journalists around the world.

In terms of the narrative thrust of these stories, the WHO initially acknowledged that PHC represented a new and bold departure from its previous focus areas, and required a different level of framing and thinking about the causes of and responses to health problems. The challenge was summed up in the introduction to a special issue of *World Health* released in April 1975 to coincide with the discussion of PHC at the World Health Assembly a month later. In the opening pages, Kenneth Newell stressed that the picture across the world was ‘one of quiet sorrow and helplessness rather than of emergency or dire tragedy…. Such a picture is not of earthquake, flood or famine. It is a *different type of drama* [emphasis added]; a family disaster with a built-in inevitability; a low-key event which one can see will be repeated and repeated unless some radical change of direction is initiated’.^45^ Newell’s point was that the consequences of disease outbreaks, natural disasters or famine could be immediate, more easily comprehensible and required solutions which could be readily envisaged if not provided. However, the problems that PHC sought to tackle were more ambiguous. In a culture supposedly accustomed to the glamour of technology and eradication programmes, how could support for much less noisy basic services be generated?

The initial response centred on a standard WHO trope of ‘before and after’ photographic narratives. These were used to present PHC as a hopeful but realistic vision of the future by contrasting it to a bleak past of inequality and misery. To emphasise this reading, the people shown to signify ‘before’ PHC looked beaten down and mournful; some howled in apparent anguish at their plight. Occupying the first half of the April 1975 special issue, such representations were contrasted to a more orderly ‘after’ picture making up the second half, which was brighter by comparison and showed happy, prosperous communities. Relatively simple, such a visual depiction of the benefits wrought by adopting PHC contributed to the intended reading of progression towards an incontrovertibly better life and the idea that communities, in time, would take the initiative to help themselves. Although conducted under the guise of sober reporting, with the camera held to convey evidence of conditions around the world, this particular display was loaded with assumptions and a good deal of expectation regarding what PHC could help to achieve. This was designed to influence contemporary mindsets; it represented a purposeful move by the WHO, deployed in the context of a political construction of communities as visible objects.

As recorded in the WHO’s ‘Third Ten Tears’, by 1977, having reviewed earlier public information activities, a WHO working group nevertheless concluded that the message of PHC had not come across in a sufficiently comprehensive way. Too much emphasis on rural development had caused confusion and given the impression that PHC was only for rural communities. Multiple understandings continued to exist within the WHO itself. An in-house survey claimed that ‘no-one connected PHC with affluent countries or with self-care’, and that the common denominator seemed to be that of the ‘organization of services at the lowest echelon including delivery of health care by personnel trained in the field’.^46^ This meant that the public information activities associated with PHC needed to be overhauled, especially given that they were targeting a diverse audience comprising decision-makers, health professionals, health institutions and the lay public. The WHO acknowledged the need to unite around a shared direction and a common picture. Nonetheless, even by the late 1970s a detailed view of what exactly this picture looked like was still in flux.

The Alma-Ata conference was envisioned as a high-profile opportunity to advance the PHC vision. For the WHO, it was to be a ‘trigger mechanism’ to advocate the idea of community participation by showing it in action, and portraying it as attractive and achievable when the audience was thought to be receptive.^47^ A public information component was therefore sanctioned as part of the conference preparations. Success stories and model examples were selected to help promote the concept of PHC to member countries where programmes were non-existent, and address its perceived status as short-term or second-rate. At the Palace of Lenin, the Alma-Ata conference venue, provisions were made for journalistic coverage through a radio studio and dedicated telephone and telex services.^48^ Information kits were distributed and short radio interviews with people involved in PHC delivery prepared. In addition, the Palace’s foyer space was requisitioned for an extensive WHO/UNICEF photographic exhibition, with issues of *World Health* containing PHC-related photo-stories being made available to delegates.

The Palace’s theatre was reserved for film screenings: fifteen films, contributed by national governments, were selected by a steering group which graded them for their depiction of innovative aspects of PHC, potential replicability in other countries, creativity and teaching excellence.^49^ The films showed a variety of PHC activities from around the world, including: communities in Mexico which identified their own priorities and satisfied their health needs; self-reliance in Vietnam; and how a medical school in India had made service to the community a part of its curriculum. With regard to style, Kirsten Ostherr describes how another film in this series, about Mozambique, adopted ‘direct cinema’, shooting scenes to give viewers the impression of being a participant rather than an observer, aided by the use of local sounds and the singing of village health workers. This was in contrast to earlier films, which had used exotic mood soundtracks featuring, for instance, native drumming.^50^ The films were presented as giving viewers a sense of the reality, making the audience feel as though they were actually in those locations. The aim was not only to elicit an emotional response of empathy, but also to present evidence of the good work being carried out. Ultimately, however, these were highly scripted and prescribed versions of reality which had been sanctioned by the steering group.

In the photographs, community health workers increasingly appeared as a central visual reference point for PHC. The essential role described by the term ‘community health worker’ had existed for a long time in many countries around the world.^51^ International health institutions in the twentieth century recognised that there were situations which required using auxiliary workers drawn from communities, the idea receiving particular attention in the interwar years when the League of Nations Health Organization (LNHO) considered these workers in the context of an emphasis on rural health. At the 1937 Conference on Rural Hygiene, held in Bandung, Indonesia, the LNHO recommended the utilisation of non-medical health personnel in areas underserved or not covered by health services, in tandem with an increased attention to community context, namely indigenous languages, cultures and traditions.^52^ In the post-war period, many countries suffered from, or anticipated, a shortfall in trained personnel and, in other cases, a movement of qualified professionals to other countries or private practice, thus emphasising the need for a strong CHW cadre. Auxiliary health workers could include primary school teachers, members of the police force, volunteers and untrained personnel. As well as drawing on the availability and cost effectiveness of training ‘local people to do relatively simple local jobs’,^53^ it was hoped that this would inspire local confidence and build genuine community participation in the long term.

In the years leading up to the Alma-Ata conference, the CHW model was presented as having great potential, be it in the form of ‘village health teams’ to address issues of rural health, or ‘medical assistants’ to help meet the needs of communities.^54^ The conference itself was envisioned as a pivotal moment in harnessing and directing the power of CHWs on a larger scale, with a view to enhancing the visibility of PHC. Point four of the Alma-Ata Declaration affirmed that people should have the right and duty to participate in the development and direction of a community-responsive health care system, and recommended the creation of more CHW programmes.^55^ The message did not advocate leaving people to it; when specialist advice or more complicated care was needed, for instance, the CHW would ideally be able to turn to more highly-trained staff.^56^

CHWs were selected as key protagonists in the WHO’s new visual strategy, and presented as inextricably linked with the push towards PHC. Photo stories which featured them moved away from ‘before and after’ narratives to develop more intricate themes. Stories presented a new range of heroes and were characterised by tales of courage and determination. This was reinforced by familiar narratives which emulated the standard conventions associated with doctors and other medical professionals, such as the position of CHWs in relation to the patient suggesting a power dynamic, and their professional accoutrements marking their skill as well as the medical tradition into which they were supposed to fit. Photographers used light, shadow and perspective to further emphasise this reading and to develop an attractive vision which matched the aspirations of the Declaration.

**Figure 1:**
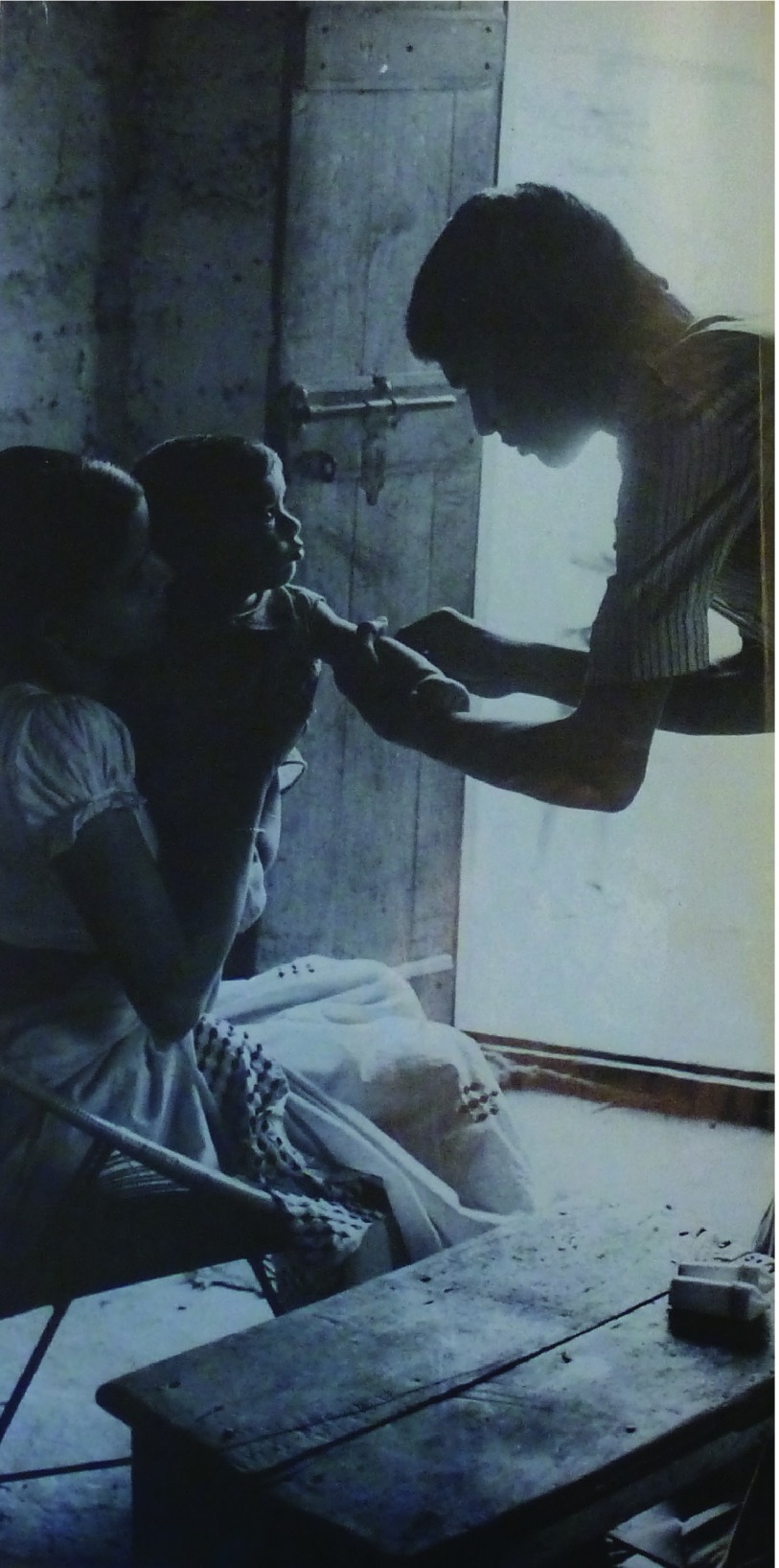
‘Millions of people are still deprived of health as a human right. But the means exist to bring this right closer to reality, and at a cost those millions can afford’, *World Health*, May 1978, page 2. © WHO/A.S. Kochar.

Figure 1 is a case in point. This was the first photograph which greeted readers of *World Health*’s PHC special issue released in May 1978 to coincide with World Health Day. The caption does not reveal specific details about the health worker or the family pictured. Instead, the scene is used to symbolise the ideas put forward in the accompanying caption: ‘Millions of people are still deprived of health as a human right. But the means exist to bring this right closer to reality, and at a cost those millions can afford.’ The photograph was therefore meant to embody the practicalities and aspirations of PHC. It pictured the health worker against sunlight flooding in through the open door, giving him an ethereal presence. In terms of the composition and the relationship of those in the image, the child and mother literally look up to the health worker (and the viewer is meant to infer this figuratively as well). In addition to making use of proximity and the ‘healing touch’, this photograph also pictured the CHW bending to serve, suggesting humility. The expressions on their faces are either partially obscured or neutral, thereby being somewhat open to interpretation of the viewer. However, the way that the child’s gaze fixes on the worker is a crucial and central point of the image. It does not suggest worry or hurt at the action being performed; it is serious, but trusting and interested.

The theme of saviour is further enforced by the use of light and shadow. It appears as if the child is being brought ‘into the light’, signalling a more hopeful and brighter existence thanks to this intervention. While there is only rudimentary information as to the precise location, this scene seems to take place in the home: the mother is seated and the worker stands as if he has just entered. In this context the open door fulfils another function, suggesting that the worker’s presence is welcome, and that light, signalling hope, has been permitted into the home. These themes were echoed in other WHO photographs, such as the one selected for a piece attributed to Halfdan Mahler in the *Public Health Reports* publication: the photograph ‘Community health worker in rural India makes home visit to provide prenatal care’ showed the CHW bending over the pregnant mother’s bed, with one hand holding hers and the other gently resting against her cheek. Again, light picked out the mother’s expectant gaze, which was concentrated fully on the health worker.^57^ Cases such as this one illustrate that, with regard to PHC, the WHO continued its drive to place photography into non-agency outlets as a means to further spread the message.

While there may have been a degree of path-dependency from previous campaigns, the photographs also represented a very conscious effort to secure the trust of local populations, thereby confronting the widespread assumption that CHWs provided temporary or second-rate solutions. While there were instances when individual CHWs were picked out and their story told, in many other cases the individual was left in shadow to suggest an every-person, an unremarkable individual who did remarkable things. China’s barefoot doctors provided inspiration in this context. *World Health* focused on the ‘million-strong army of peasant doctors’ who were ‘probably the most important example of community action in the fight for good health in China’.^58^ Indeed, there were overlaps between the WHO’s photographic renditions of CHWs and Chinese posters of barefoot doctors. Pang’s analysis shows that the latter were not depicted with clenched fists, frowning faces or piercing stares; they were neither assertive or threatening.^59^ Instead of being political figures, CHWs calmly got on with the job, showing a quiet dedication rather than a revolutionary spirit. These choices were reflected in the WHO’s photographs which presented CHWs as calm and placid individuals, pursuing a quiet dedication to their appointments. Even when the physical exertion of this work was mentioned in the accompanying text in the photo stories, it was largely absent in the photographs.

Proximity was an oft-repeated theme. CHWs had a clear advantage in this respect, since the community-centred model challenged predominant views of clinical relations based upon hierarchy and distance between the patient and the health professional qualified to dispense care. When representing CHWs in photo stories, as above, the WHO placed great importance on physical contact and touch, emphasising the healing hands of CHWs, extended and quite literally reaching out, but also depictions of domestic settings which showed the ability of CHWs to deliver care inside the household. Trusted access to the homestead helped underscore CHWs as ‘of the people’, able to accomplish certain duties with greater ease and acceptance. *World Health*’s PHC issue of April 1975 included a photograph of an auxiliary health worker calling on a village community in West Azerbaijan, and was typical of how these different themes were conjoined.^60^ Both worker and community member were displayed fully in focus in an animated but relaxed conversation, in a modest but neat room. Kneeling suggested humility and a lack of hierarchy, but the pen and jotter provided an air of professionalism and diligence. Such renditions shared parallels with past photographic series. Exploring photographs of nurses in the 1930s, Shawn Michelle Smith has identified the importance of the threshold in photographs which represented the nurse as ‘the minister and messenger of health, bringing the private home into conformity with the health of the larger community’.^61^

The WHO also pictured larger community spaces, many of which included CHWs imparting knowledge or being listened to reverentially by community members. Sometimes this was presented in the style of a lecture, with the audience seated and looking up at the instructor, but in many instances the different actors were presented as being on the same level, or in a circle, discussing together. Again, this spoke to the theme of proximity and non-hierarchical relationships, showing that CHWs performed functions that allowed them to approach problems through dialogue and cooperation with the community. In each case, viewers were meant to be unconscious of the photographer’s presence, giving the impression that they were afforded unrestricted and privileged access to these situations. Unlike the earlier style of photography, where community members appealed to the viewer by engaging the camera lens directly, in the photographs described above the viewer saw evidence of community members helping themselves. However, like much of the WHO’s photographic repertoire, these were highly authored and in some cases scripted compositions, with the published variants commonly selected in preference to dozens of similar variants which, having been assembled and weighted in terms of their effectiveness, were consigned to the cutting room floor.^62^

**Figure 2:**
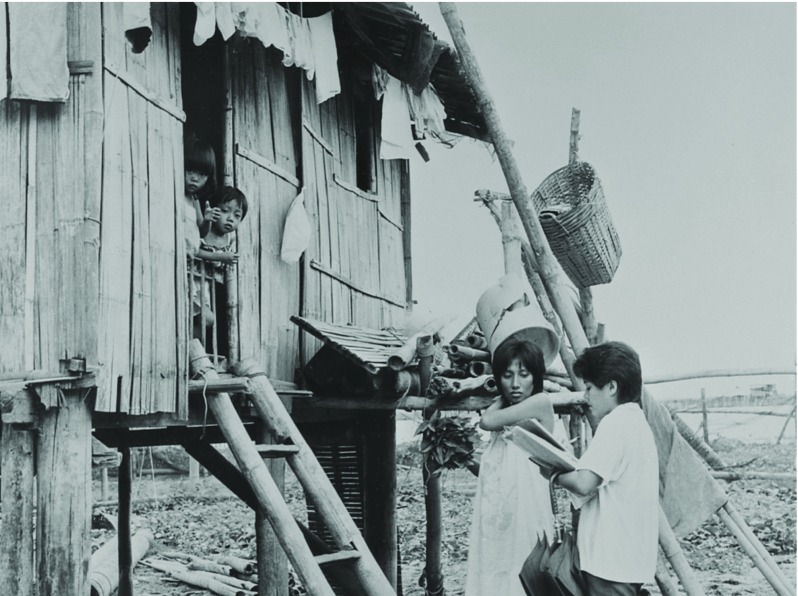
‘A community health worker on her house rounds’, (1980). © WHO/Ghulam Zafar.

An attendant focus on mothers and children in the household showed who was benefitting from PHC. This emphasised the crucial role of the CHW by extension, but was also used to strike an emotional chord and add the sought-after ‘drama’. In the outwardly straightforward photograph of a CHW on her house rounds, which appeared in the January/February 1988 number of *World Health* (Figure 2), there were many sub-narratives. The pristine white shirt, umbrella and files carried by the CHW convey a sense of order being brought to the rather ramshackle house with its makeshift and weather-worn wood. Nevertheless, the scene of domesticity would likely have held universal appeal despite viewers’ different backgrounds. The scene takes place on washday, with baskets, buckets and drying clothes hung from the roof. Mother has taken time from this routine to meet with the CHW. The children, sequestered indoors, display a childish inquisitiveness, craning their heads to observe the encounter (and most likely also the presence of the photographer, Ghulam Zafar). For many years WHO’s photographic repertoire had focused on the mothers and children (as did UNESCO and UNICEF), and it is therefore interesting that similar presentations were retained. Even though visual representations of PHC spoke to a sense of departure from old conventions by advancing the idea of communities helping themselves, they recalled an established visual tradition of engaging and persuading audiences by using ideologies of rescue, care and compassion developed for Western audiences in the aftermath of the Second World War.^63^

When not pictured in the home environment, CHWs were commonly photographed on the move. Mobility was another key theme which emphasised the CHW’s perceived ability and commitment to better deliver health to underserved areas. For Madagascar, for example, it was reported that ‘On foot, on bicycles, mounted on oxen, in carts…nearly 500 of them have set forth into each of our prefectures to take up their posts’.^64^ *World Health* editors selected photographs of community workers walking, riding horses, bicycles and motorcycles, or otherwise moving through the scenery. While the distances involved could be used to emphasise dedication, this presentation also showed the CHW in the guise of bringing something new, and something worth delivering as well as receiving. Unlike the disease vector, which brought harm, this mobility delivered health. Those mounted on motor vehicles helped convey a sense of urgency, while those who travelled on foot or using animals were meant to connote persistence, as well as ‘appropriate technology’ in preference to larger and more expensive all-terrain vehicles. In each case, they helped to lend a sense of drama, as well as symbolically representing progress and dynamism. However, as Henrietta Lidchi points out in relation to later NGO-generated imagery, this reading was heavily dependent on the viewer. The grassroots, tailor-made and participatory elements that for some were positive dimensions of PHC, for others were evidence of rudimentary, technologically backward and ineffective health provision.^65^ Notwithstanding this, the frequency of repetition suggests that the WHO saw this as a useful and positive image to associate with CHWs and PHC.

Aside from the intricate focus on CHWs and the people they served, the WHO also showcased the more general benefits of community collaboration. An aspiration vocalised at Alma-Ata was that action in the form of CHW programmes would inspire further community collaboration and translate into broader community development. The ‘community’ was therefore also made a prominent actor in the photographs. Sometimes this was straightforward, as when a large group (ten or more, arranged informally or tightly packed together) engaged the camera directly. This was a standard and effective technique: smiles signified happiness and connoted that community schemes were not only valuable and effective but were preferable to those they served. The inclusion of many people also suggested that this happiness was felt throughout the community. Many more photographs captured community industriousness in reportage style, as groups bowed their heads and raised their tools. These were meant to look un-posed and unscripted, capturing and presenting the endeavour ‘live’ (even though, as photographic mission reports and contact sheets confirm, they were often highly staged). Other shots displayed evidence of successes, such as damming of rivers to improve rice production and a productive paddy field with a newly constructed irrigation system. In this sense, they shared further similarities with Chinese posters, which did not show the barefoot doctor advocating the struggles typical of the Cultural Revolution, but pictured as the caretaker of the village.^66^ *World Health*’s pages frequently contained this sort of ‘evidence’, which was also well-represented elsewhere: Mahler’s *Public Health Reports* article featured a prominent photograph that showed a protected well being constructed according to the instructions of a CHW in the village of Khariz, Iran.^67^

Such representations were attempts to address the fact that some elements competed against established (and attractive) narratives and were also more difficult to represent visually, such as the hope that community participation would embolden some greater community spirit. To this end, photography relied heavily on symbolic connotations and the interplay between text and image. This active visual style supported the air of immediacy, to suggest that work towards these goals was going on as the viewer browsed the pages. Other representations reported on ambitious development activities that had come from ‘humble beginnings’, or measurable successes in terms of children immunised against whooping cough, measles, diphtheria, poliomyelitis and TB, and provision of clean drinking water. Throughout the late 1980s and early 1990s, in articles such as ‘Harmony at the Village Level’,^68^ ‘Wisdom and Guidance from the Community’^69^ and ‘Helping Oneself to Health’,^70^ the WHO forwarded a hopeful and optimistic narrative that learning from experiences and pulling together could overcome ingrained challenges and deliver significant gains where other strategies had persistently failed.

In sum, in order to promote PHC, the WHO recognised the need to inspire – that is, to elicit an emotional response – and not simply seek to convince by way of logic or a rational argument. The WHO sought to shape the affective and moral landscape that had hitherto relegated PHC to a position of lesser importance. Experience from previous campaigns recommended that photo stories, films and exhibitions should not simply provide information, but actively seek to change mindsets by being attractive and captivating, humanising the information and making it relevant and appealing to different audiences.^71^ The WHO’s representations of CHW programmes relied upon key themes of professionalism, proximity, mobility and community development. The agency sought to make CHWs visible as reliable and trustworthy, close-to-community elements that could bring healthcare to the most remote households. Importantly, CHWs were also community members that assumed a prominent role in mobilisation, seeking to inspire others to contribute to development. Notwithstanding this, WHO efforts at enhancing the visibility of CHWs faced significant challenges almost immediately.

## 4 Media Challenges in an Unfavourable Political Context

The considerable efforts to publicise CHWs and the PHC approach took place in an environment of mounting pessimism and criticism of Alma-Ata’s goals, which were said to be unrealistic and idealistic.^72^ The rival concept of Selective Primary Health Care (SPHC) effectively ‘took the decision-making power and control central to PHC away from the communities and delivered it to foreign consultants with technical expertise in these specific areas’.^73^ SPHC focused on a limited number of key medical interventions that were considered more cost-effective, and the impact of which could be easily monitored and evaluated.^74^ These interventions included growth-monitoring, oral rehydration therapy, breastfeeding and immunisation – and thus the approach also became known under the acronym ‘GOBI’.^75^ Overall, the SPHC vision, translated into GOBI, meant a shift away from the community emphasis and CHW programmes, which were often tasked with responsibilities and functions that were hard to measure and quantify. At the same time, economic recession in the 1980s brought shifts in the policy environment as decolonisation, democratisation and self-reliance were replaced by World Bank-driven policies of structural adjustment emphasising quantification and bureaucratisation.^76^

CHW programmes were among the first to suffer in this climate, but many also experienced planning and implementation problems. In 1980, a meeting convened by the WHO and UNICEF in Kingston, Jamaica, to collect, share and analyse CHW information and experiences identified the need for more information from those with knowledge of mobilising communities, and particularly in the selection, support and evaluation of the use of CHWs.^77^ But there were many other challenges. To begin with, and despite the WHO’s media effort, there continued to be fundamental misunderstandings about the very idea of PHC. A 1982 ‘Review on Primary Health Care Development’ in the eastern Mediterranean region also reported that the lack of understanding about the true nature of PHC through community involvement extended to decision-makers, health planners and health practitioners. The review drew the sombre conclusion that although community involvement figured in legislation, country documents and training curricula, rarely was it practised in reality.^78^ It noted a conspicuous gap between idealised models and the practice of community involvement.

Furthermore, and contrary to what was envisioned, workers drawn from the communities were not universally accepted. In 1983, an inter-regional study into CHWs highlighted common weaknesses in selecting the workers.^79^ Although done by community leaders, selection commonly happened at a time when the community as a whole had only a partial understanding of the CHW’s supposed role, meaning that the individuals chosen were not always suitable candidates. Lack of understanding engendered low interest, poor involvement and unhelpful expectations. Another, more fundamental, implementation challenge was that some communities lacked a tradition of sustained cooperation.^80^ Furthermore, community-centred programmes quickly had to grapple with the complex political realities on the ground. In particular, they had to face the fact that community development initiatives very often led to the entrenchment of existing power structures and inequalities instead of contributing to increased participation and democratisation. Daniel Immerwahr has shown how post-war community development initiatives were predicated upon a particular vision of the rural world, ‘a fantasy that the communities in question were essentially neighborly places that only required a nudge to undertake the therapeutic and cooperative processes of self-improvement’.^81^ This romantic view depoliticised the communities in question, overlooking ‘power within communities and power relationships between communities and the larger societies around them’.^82^

Failure to meet expectations was another problem, and one that was again linked to the media image. In the wake of the Alma-Ata Declaration, it was reported to the WHO that large sections of rural populations previously underserved had been led to expect adequate and competent coverage through CHW schemes. The goal of inspiring attention and belief among the underserved had been achieved, but in cases where even the ‘poorest of the poor’ were assured that alleviation of their miseries was possible, failure to meet these expectations was said to be destroying the image and credibility of the approach.^83^ Just as earlier promises of health gains driven by technological interventions had, over time, drawn criticism, the failure to deliver community programmes created similar reactions. To the forefront of this was a concern that the representation of these programmes by the WHO may have suggested more was being done to address underlying problems than may actually have been the case in certain areas. To borrow Mark Nichter’s phrasing, people risked becoming ‘anesthetised’ by hopeful and repetitive narratives, which may have matched their aspirations but not their experiences.^84^

During the 1980s, the WHO sought to push back against this inhospitable environment, at first by way of a series of discussions and consultations, which included reflection on the crucial role of the media. An inter-regional conference held in Yaoundé, Cameroon, in 1986 reiterated the role of CHWs as pillars of the Alma-Ata Health for All vision.^85^ Opening the conference, Halfdan Mahler delivered a rallying call drawing upon the benefits of community involvement, but optimism did not help when decentralising responsibility to communities ran counter to the beliefs of some health decision-makers, or posed risks to non-democratic governments.^86^ In the communities, lack of cohesion, social stratification, the scattered nature of populations and problems in the selection of health workers all limited the success of programmes.^87^

Despite agreement on their importance, much remained to be done to implement CHW programmes effectively. An initial problem was the very definition and designation of CHWs. The Yaoundé conference forwarded one of the most enduring and widely-held definitions for CHWs: that they should be ‘members of the communities where they worked, be selected by and answerable to the communities, be supported by the health system but not necessarily a part of its organisation, and have shorter training than professional workers’.^88^ But the term ‘community health worker’ was itself an issue at these meetings. Although the WHO used it as a generic term, different countries used a range of names, including family welfare educator (Botswana), rural doctor and health aide (China), community health agent (Ethiopia), community health guide (India), community health aide (Jamaica), village health worker (Nigeria) and barangay health worker (Philippines).^89^ This added to the cacophony.

The report of the WHO’s ‘Strengthening the Performance of Community Health Workers in Primary Health Care’ working group, published in 1989, showed that problems persisted, and were in some cases mounting. It noted that in the initial enthusiasm for PHC, policies had been hastily devised leading to serious weaknesses in implementation. The energy with which it had been promoted and the moral force of the arguments obliged countries to demonstrate their commitment to it, with CHW programmes seen as the easiest, cheapest and most obvious ways for governments to do so. In addition, many countries had defined the functions of CHWs on the basis of the elements of PHC contained in the Alma-Ata Declaration, which constituted a very broad range of tasks indeed. The working group considered it unreasonable and unrealistic to routinely assign this array of functions to CHWs. Furthermore, there remained a misunderstanding of the highly stratified nature of communities, with class, caste and other divisions affecting the position and loyalties of CHWs, as well as public demands on them.^90^ In some cases, health planners who claimed to believe in community participation really meant that they were leaving rural and other underserved areas to themselves, a further subversive interpretation of the goals.^91^

Part of the consultation process carried out by the WHO during the 1980s naturally centred on how the media could be used to help sell and reinvigorate enthusiasm for the approach. At a 1981 WHO/UNICEF regional workshop on information, education and communication on health, the health and science editor of Manila’s *Times Journal*, Alberto Rous, summarised the challenges faced by the PHC approach. Rous highlighted the variations of the individual countries and their media profiles, but also the necessary shift in emphasis from the sophisticated medical technology and infrastructure which dominated health matters in the media, to items dealing with the provision of basic necessities and appropriate technology. He also noted the danger of oversell, which could raise expectations, false hopes or even panic in extreme cases. He concluded that selling a ‘relatively new and radical concept like PHC, which would mean persuading people to assume responsibility for their own health and well-being, may yet turn out to be one of the biggest and most difficult selling jobs for mediamen in this century’.^92^

Participants at a 1983 media seminar on ‘Health for All Through Primary Health Care’ argued that news about PHC would sell if packaged attractively, but information needed to go hand in hand with service.^93^ In 1985, a meeting of the Consultative Group on the Organization of Health Systems Based on Primary Health Care observed that many countries were still facing the problem of explaining and promoting PHC to target groups such as politicians, health professionals, health-related groups and the public at large. The recommendation was that PHC should be presented as being a part of first-class medicine, and as a declared national aspiration. ‘For the politicians and the public at large, the ministry of health should use every opportunity and means, both modern and traditional, to create awareness and understanding of PHC.’ It also advised considering commercial marketing and mass communication to ‘sell the idea’ alongside more traditional forms of communication.^94^

The workshops therefore identified a supplementary set of challenges, which hinged directly upon the contemporary media campaigns. There was a disconnect between the aspirations of those involved in planning and the situation as represented in the WHO’s media organs. For some, the ‘one size fits all’ message suggested by model examples was unhelpful. Despite the focus of the articles in *World Health*, Dr Daniel Flahault, chief medical officer for training of auxiliary personnel in the division of Health Manpower Development, admitted that ‘we do not believe…there is a model which can fit the needs of many countries’. He advocated instead that the solutions adopted by a given country could serve as an example for adaptation.^95^ There was also the criticism that, in attempting to prove and sell the CHW concept, films and photo stories put forward a vision that was too idealistic, which presented few real life struggles and gave an impression of a ‘neatness and symmetry’ that was lacking on the ground.^96^ While it was recognised that the stories did not cover flawless systems, ‘where everyone works in harmony, without personality clashes, causing integrated development to appear as if by magic throughout the land’,^97^ they were naturally geared towards inspiring uptake and so obstacles were played down. To achieve its goals, the WHO presented programmes in their best light, and the selection and arrangement of visual content was meant to discipline the gaze and inspire audiences to certain, generally positive, conclusions about community participation. Yet this was the paradox of the image: while the idealised image was held to inspire, a realistic image to demonstrate problems or challenges, which would have been accurate and potentially useful, could also prove detrimental.

Media campaigns also faced more practical obstacles. As the years passed, it proved difficult to procure relevant and interesting imagery in support of the WHO’s messages. In preparation for a photo story on the People’s Republic of Mongolia, for instance, Didier Henrioud, one of the WHO’s photographers based at its Geneva headquarters, was dispatched to photograph health institutions and activities in the country suitable for an exhibition and articles on health services in *World Health*. The Director of the Department of Information, Mr C.W. Morrow, recommended that this assignment ‘should not be carried out only in large institutions in the Capital city, but also in the field, where extremely interesting opportunities are offered to show rural health/PHC activities’.^98^ However, Henrioud’s travel report noted that he worked under the constant supervision of two ministry officials, who only permitted him to photograph the more sophisticated aspects of health activities in the country^99^. The freelance photographer Paul Almasy recalled similar experiences in a retrospective piece for *World Health* in 1990: frequently he too was shown only modern facilities.^100^ Such problems entrenched the reliance on model examples where the message was clear and to the WHO’s liking, but also underscored the widely-held preferences which linked ‘modern’ with ‘effective’. Overall, these challenges meant that the rhetoric and imagery of the community approach continued to face an uphill struggle against the established idea of Western medicine as the epitome of what an advanced health system should look like. The authority of Western medicine continued to exert an enormous influence over perceptions. The result was a message which was somewhat of a compromise, and which therefore failed to capture the attention or excite the diverse audiences. Ultimately, this influenced the willingness to organise new programmes or when choosing between approaches.

By the end of the 1980s, the WHO was still uncertain about how best to present the PHC concept, and was facing difficulties in putting the message across effectively. These problems persisted into the 1990s when community participation was once again the central theme of the 1994 World Health Assembly. As was customary, the media teams sought to create a composite video and complementary photograph exhibition for presentation during the Assembly’s technical discussions,^101^ but familiar questions continued to be burdensome. These pertained to audience (policy-makers, social groups or local leaders), purpose (to expand community action for health, or show small successes to inspire action) and even content, with the photographer Tibor Farkas admitting that he had little good material that had not already been used.^102^ Problems such as these entrenched the reliance on model examples and ultimately restricted the view. In the same year, a WHO report concluded that there was an enormous gap between the acceptance of the principles of global solidarity at the Alma-Ata conference and subsequent conferences and the actual implementation of those principles.^103^ The political context – pertaining to both international factors and the micro-level obstacles to implementation – ultimately decided the fate of the visual campaign that had sought to advance PHC.

## 5 Conclusion

This article explored the ways in which the WHO sought to advance the PHC agenda around the 1978 Alma-Ata conference, in particular by focusing on CHW programmes. Photography was believed to be a privileged tool to change widely-held assumptions and to inspire community action, and was thus used by the WHO to help overcome challenges relating to the implementation of community-centred approaches and PHC more generally. Photographic narratives were mobilised by the WHO to build up a visual vocabulary of community action for health. They were used to show readers what was going on, to help them imagine the essential role of CHW programmes and to show how such programmes might be enacted. However, the point was less about capturing reality than presenting a certain narrative in an understandable and captivating manner – thus seeking to shape the political and moral imagination of decision-makers, donors and the public. Stylistically, these images negotiated between traditional narratives and novel representations. Key themes in these visual representations were identified: professionalism, proximity, mobility and the idea of CHWs as facilitators of community development.

The Alma-Ata conference presented the opportunity to advance PHC, and CHW programmes were placed at centre-stage in this project. However, despite the great hopes surrounding the Alma-Ata vision of ‘Health for All’, there was a mismatch between visual representations and the stark realities on the ground – and here the representations put forward by the WHO may have been partly to blame. In order to ‘sell’ PHC, the WHO portrayed CHWs in a favourable light – yet it was perhaps too favourable, and difficulties not always sufficiently elaborated upon. Model examples were celebrated which did not fit the needs of everyone, or were unavailable to everyone. Photographs presented few real life struggles; complexity and tensions were glossed over. The media image also helped to disseminate the view that basic services such as CHWs could be achieved simply and easily without high cost. This was an oversimplification, and the WHO’s output ended up offering a selective visibility, which, while it helped to support the call for action, ultimately led to misunderstandings. This had repercussions further down the road, not least by creating disappointment among the public and by discouraging decision-makers and health workers. Ultimately, the picture was not consistent and appealing enough to displace entrenched images of a ‘capable’ health system – one based upon Western biomedical conceptions, the authority of doctors and nurses, specialised scientific knowledge, pharmacological solutions and technological efficacy.

The argument here presented explored in depth the visual politics of the WHO, and particularly how it changed after 1970 to accommodate a new strategy. Visual politics works in ways akin to advertising, which can be considered a strategy ‘aimed not so much at making you buy something, but rather at having you looking at certain things rather than others’.^104^ This argument provided a clear demonstration that what becomes visible in global health is the result of political processes through which particular versions of the real are presented, contested and fought over.^105^ Raising visibility is never a straightforward process of creating and disseminating pictures or posters, and prolific distribution does not necessarily bring success. While media campaigns have become increasingly essential in public health work across the second half of the twentieth century, it would be erroneous to overlook the political context in which these campaigns occur. Considering the politics of visuality, and not simply visual representations as abstracted from the context of their production and dissemination, allows one to investigate the reception of messages, which are never absorbed uncritically in line with the creator’s intentions. With this focus on visual politics, the article thus supplements the study of problem-definition in global health, thus encouraging broader reflection on the visual politics surrounding health issues and programmes at an international level.^106^

The article also speaks to the relative neglect of the history of CHW programmes in the academic literature. By and large, the scholarly comment on CHWs is limited to contemporary examples and to the assessment of the more recent past.^107^ CHW programmes continue to be championed as a way to guide health system reform in middle-income countries, emerging states and also some developed nations.^108^ However, the history of CHW programmes, and particularly the standpoint of the WHO therein, has remained largely understudied. This gap is important because, while CHWs are ubiquitous and highly visible to the communities they serve, undertaking many essential roles which would otherwise not take place, they are paradoxically an ‘invisible cadre’ at the levels of policy, strategy and financing.^109^ It is therefore important to consider their history in order to be able to raise their profile in the present.

The visual politics approach is significant also because it offers important clues to identify existing potential for change. One reason that explains the lack of success of WHO representations, and the ways in which they unwittingly contributed to the neglect of PHC and CHW programmes, is the fact that they were overwhelmingly top-down, without appropriate consultation and ownership by CHWs themselves. Representations that are more cognisant of these challenges may succeed where previous ones faltered. A recent profusion of photography projects and competitions have sought to encourage reflection on the role and work of CHWs. The specific intentions of these competitions vary, but tend to convey a wish to inspire action, put an end to health workforce crises, capture everyday stories and challenges, question commonly-held assumptions and demonstrate the breadth of community-led health activities.^110^ There is, therefore, a widespread belief that images can be powerful tools for changing the political and moral imagination around a particular issue. Visual representations are always political interventions, susceptible to constraints but also potentially able to push back against existing limits. In the case of CHWs, attempts at raising visibility have so far fallen short of their objectives – with detrimental effects upon the health of vulnerable groups. Nonetheless, visual representations still have an important role to play in creating the conditions for more equitable and universal access to health care. Moreover, these representations can also be, in and of themselves, exercises in the democratisation of health, by giving voice to people and perspectives that have been silenced or ignored for far too long.

## Footnotes

1.

Margaret Jones and Chandani Liyanage , ‘Traditional Medicine and Primary Health Care in Sri Lanka: Policy, Perceptions, and Practice’, Asian Review of World Histories, 6, 1 (2018), 157–184.

2.

Marcos Cueto , ‘The Origins of Primary Health Care and Selective Primary Health Care’, *American Journal of Public Health*, 94, 11 (2004), 1864–1874; Socrates Litsios, ‘The Long and Difficult Road to Alma-Ata: A Personal Reflection’, *International Journal of Health Services*, 32, 4 (2002), 709–732; Theodore Brown, Marcos Cueto, and Elizabeth Fee, ‘The World Health Organization and the Transition From “International” to “Global” Public Health’, *American Journal of Public Health*, 96, 1 (2006), 66–68; Nitsan Chorev, *The World Health Organization Between North and South* (Ithaca: Cornell University Press, 2012), 66–79.

3.
See, for example, the special issue, ‘Cancer in the twentieth century’, in *Bulletin of the History of Medicine*, 81, 1 (2007); the contributions in Friedenback and Rodogno, *Humanitarian Photography: A History* (Cambridge: Cambridge University Press, 2015); Laura Briggs, ‘Mother, Child, Race, Nation: the Visual Iconography of Rescue and the Politics of Transnational and Transracial Adoption’, *Gender and History*, 15, 2 (2003), 179–200.

4.

Mark Alleyne , *Global Lies?: Propaganda, the UN, and World Order* (Basingstoke: Palgrave Macmillan, 2003); Tom Allbeson, ‘Photographic Diplomacy in the Postwar World: UNESCO and the Conception of Photography as a Universal Language, 1946–56’, *Modern Intellectual History*, 12, 2 (2015), 383–415.

5.

Thomas David and Davide Rodogno , ‘All the world loves a picture: The World Health Organization’s visual politics, 1948–73’, in Heide Fehrenbach and Davide Rodogno (eds), *Humanitarian Photography: A History* (Cambridge: Cambridge University Press, 2015); Alexander Medcalf, ‘Between Art and Information: Communicating World Health, 1948–70’, *Journal of Global History*, 13, 1 (2018), 94–120.10.1017/S1740022817000304PMC603442829997674

6.

Medcalf, *ibid.*, 94–120.10.1017/S1740022817000304PMC603442829997674

7.

Davidand Rodogno, *op. cit.* (note 5).

8.

*Ibid*., 231–6.

9.
See Bert Hansen, *Picturing Medical Progress from Pasteur to Polio: A History of Mass Media Images and Popular Attitudes in America* (New Brunswick, NJ: Rutgers University Press, 2009), 230–52.

10.

Marcos Cueto , *The Value of Health: A History of the Pan American Health Organization* (Washington, DC: Pan American Health Organization, 2007); Randall Packard, *A History of Global Health: Interventions Into the Lives of Other Peoples* (Baltimore: Johns Hopkins University Press, 2016).

11.

Xun Zhou , ‘Reconsidering the Barefoot Doctor Programme’, Fudan Journal of the Humanities and Social Sciences, 9 (2016), 41.

12.

WHOand UNICEF, Primary Health Care: A Joint Report by the Director-General of the World Health Organization and the Executive Director of the United Nations Children’s Fund (Geneva: World Health Organization, 1978), 30–33. http://apps.who.int/iris/handle/10665/39225.

13.
The studies by David and Rodogno, and recently by Medcalf, concentrate on the WHO’s activities in this regard prior to 1970.

14.

World Health Organization, (1946) ‘Constitution of the World Health’ Organization.

15.

World Health Organization Division of Strengthening of Health Services, Strengthening of Health Services: WHO’s Response to the Changing Needs of Countries(Geneva: WHO Division of Strengthening of Health Services, 1994), 5 http://apps.who.int/iris/bitstream/10665/59543/1/WHO_SHS_94.2.pdf.

16.

Packard, *op. cit.* (note 10), 123–4.

17.

World Health Organization Division of Strengthening of Health Services, *op. cit.* (note 15), 11–12. For a take on the vertical/horizontal divide, see Valeria Oliveira-Cruz, Christoph Kurowski and Anne Mills, ‘Delivery of Priority Health Services: Searching for Synergies Within the Vertical Versus Horizontal Debate’, *Journal of International Development* 15 (2003), 67–86.

18.

Kenneth Warren , ‘The Alma-Ata Declaration: Health for All by the Year 2000?’, Britannica Book of the Year 1990 (Chicago, IL: Encyclopaedia Britannica, 1990), 26.

19.

WHOand Marcolino Candau , The Work of WHO, 1966: Annual Report of the Director-General to the World Health Assembly and to the United Nations (Geneva: WHO, 1967), vii http://www.who.int/iris/handle/10665/85795.

20.

WHO, Research in the Organization of Community Health Services(Geneva: WHO, 1972), 2 http://apps.who.int/iris/bitstream/10665/145456/1/WHA25_6_eng.pdf.

21.
See Daniel Immerwahr, *Thinking Small: The United States and the Lure of Community Development* (Cambridge, MA: Harvard University Press, 2015), 166–77.

22.

Walter Bruchhausen , ‘From Charity to Development: Christian International Health Organizations, 1945–78’, Hygiea Internationalis, 13, 1 (2016), 127–128.

23.

*Contact*, June 1972.

24.
In 1974 the UN requested all its specialised agencies to develop action plans in light of the NIEO. See Chorev, *op. cit.* (note 2), 54.

25.

Socrates Litsios , ‘The Christian Medical Commission and the Development of the World Health Organization’s Primary Health Care Approach’, The American Journal of Public Health, 94, 11 (2004), 1890.10.2105/ajph.94.11.1884PMC144855515514223

26.
Mahler’s background was in tuberculosis and community work, after which he was appointed chief of the Tuberculosis Unit at the WHO’s headquarters. As Marcos Cueto describes, he also directed the WHO Project on Systems Analysis, a programme working to improve national capabilities in health planning. Cueto, *op. cit.* (note 2), 1865.

27.
SHS/Reg.Adv.74/4 Meeting of Regional Advisers on Community Health Services, Geneva 29 April–3 May 1974. WHO Archives.

28.

Kenneth Newell , Health By the People (Geneva: World Health Organization, 1975), xii.

29.

*Ibid*., 193.

30.

V. Djukanovic and E.P. Mach (eds), Alternative Approaches to Meeting Basic Health Needs in Developing Countries: A Joint UNICEF/WHO Study (Geneva: World Health Organization, 1975), 7.

31.

*Ibid*., 105.

32.

O. Adeniyi-Jones , ‘Community Involvement: New Approaches’, WHO Chronicle, 30 (1976), 8–9.1246879

33.
‘Human Investment in Environmental Sanitation Programmes’, WHO Chronicle, 30(1976), 26.1246874

34.

WHOand UNICEF, Primary Health Care: Report of the International Conference on Primary Health Care, Alma-Ata, USSR, 6–12 September 1978 (Geneva: World Health Organization, 1978), 11 http://www.who.int/iris/handle/10665/39228.

35.

WHOand UNICEF, *Primary Health Care: A Joint Report*, 32. http://www.who.int/iris/handle/10665/39225.

36.

J.H. Bryant , Health and the Developing World (Ithaca, NY: Cornell University Press, 1969), ix–x. Quoted in Cueto, *op. cit.* (note 2), 1864.

37.

WHOand UNICEF, *op. cit.* (note 34), 3.

38.

Litsios, *op. cit.* (note 25), 1892.

39.
Letter from Dr M.A.C. Dowling, Chief Medical Officer, Educational Communication Systems, Division of Health Manpower Development to Dr P.G.H. Gell, Head of Dept. of Experimental Pathology, The Medical School, University of Birmingham, UK, dated 4th May 1977. E 3/134/2 – Training of Health Auxiliaries – General. WHO Archives.

40.
Regional Director for Europe’s Draft Report, 21 March 1978, P21/87/5 WHO International Conference on PHC, Jacket 5. WHO Archives.

41.
See for instance: ‘Mit Halfa Meets the Twentieth Century’, *WHO Newsletter*, June–July 1955; ‘The Villager Meets the Twentieth Century’, *World Health*, September–October 1961, 7.

42.

WHOand UNICEF, *op. cit.* (note 35), 11.

43.

WHOand Halfdan Mahler , The Work of WHO: Annual Report of the Director-General to the World Health Assembly and to the United Nations, 1973 (Geneva: WHO, 1974), 156–157. http://www.who.int/iris/handle/10665/85868.

44.

Ibid., 166.

45.

*World Health*, April 1975.

46.
Minutes of a meeting of the Steering Committee on Primary Health Care, 27 April 1977, quoted in World Health Organization, *The Third Ten Years of the World Health Organization: 1968–77* (Geneva: World Health Organization, 2008), 298–9.

47.
Working Page on the International Conference on PHC, dated January 1977. P 21/87/5, WHO International Conference on PHC, Jacket 2. WHO Archives.

48.
Public Information Arrangements for the International Conference on PHC, dated 12 September 1978. P 21/87/5, WHO International Conference on PHC, Jacket 9. WHO Archives.

49.
WHO Telegram 3 May 1978. P 21/87/5, WHO International Conference on PHC, Jacket 6. WHO Archives.

50.

Kirsten Ostherr , Cinematic Prophylaxis: Globalization and Contagion in the Discourse of World Health (Durham: Duke University Press, 2006), 107.

51.
Prominent examples include China’s Barefoot Doctors and Russia’s Feldshers: Samuel Ramer, ‘Who was the Russian Feldsher?’, *Bulletin of the History of Medicine*, 50, 2 (1976), 213–25.779876

52.

Theodore Brown and Elizabeth Fee , ‘The Bandoeng Conference of 1937: A Milestone in Health and Development’, American Journal of Public Health, 98, 1 (2008), 42–43.1804877610.2105/AJPH.2007.119222PMC2156053

53.

Fraser Brockington , ‘The Development of Health Units’, World Health Assembly, 7 (1954), 17 http://www.who.int/iris/handle/10665/103757.

54.
‘Health Care in Rural Areas’, *WHO Chronicle*, 30 (1976), 11–17; E.J. Watson, ‘Meeting Community Health Needs: the Role of the Medical Assistant’, *WHO Chronicle*, 30 (1976), 91–6.1258464

55.

Allen L. Hixon and Gregory G. Maskarinec , ‘The Declaration of Alma Ata on its 30th Anniversary: Relevance for Family Medicine Today’, Family Medicine, 40, 8 (2008), 586.18988046

56.

WHOand UNICEF, *op. cit.* (note 37), 9–10.

57.

H. Mahler , ‘Promotion of Primary Health Care in Member Countries of WHO’, Public Health Reports, 93, 2 (1978), 107–113. https://www.ncbi.nlm.nih.gov/pmc/articles/PMC1431881/pdf/pubhealthrep00143-0005.pdf.416457PMC1431881

58.
‘Community Action Pays Off’, *World Health*, December 1975, 22.

59.

Laikwan Pang , ‘The Visual Representations of the Barefoot Doctor: Between Medical Policy and Political Struggles’, Positions: East Asia Cultures Critique, 22, 4 (2014), 842.

60.
‘An Auxiliary Health Worker Calls on a Village in West Azerbaijan’, *World Health*, April 1975, 22.

61.

Shawn Michelle Smith , ‘Nursing the nation: the 1930s public health nurse as image and icon’, in David Serlin (ed.), Imagining Illness: Public Health and Visual Culture (Minneapolis: University of Minnesota Press, 2010), 147.

62.
See Medcalf, *op. cit.* (note 5), on how this process was administered at the WHO.

63.

Briggs, *op. cit.* (note 3), 179–200.

64.
‘Health Aides of Madagascar’, *World Health*, October 1980.

65.

Henrietta Lidchi , ‘Finding the right images: British development of NGOs and the regulation of imagery’, in Heide Fehrenbach and Davide Rodogno (eds), Humanitarian Photography: A History (Cambridge: Cambridge University Press, 2015), 288.

66.

Pang, *op. cit.* (note 59), 841.

67.

Mahler, op. cit. (note 57).

68.

*World Health*, April, 1988.

69.

*World Health*, May–June, 1992.

70.

*World Health*, March–April, 1994.

71.
Such were the discussions at CESI/PC.71-17, 27 August 1971. P13/372/11, Co-ordination with UN and Specialised Agencies – World Population Year – 1974, Jacket 1. WHO Archives.

72.

Cueto, *op. cit.* (note 2), 1868.

73.

John Hall and Richard Taylor , ‘Health for All Beyond 2000: The Demise of the Alma-Ata Declaration and Primary Health Care in Developing Countries’, The Medical Journal of Australia, 178, 1 (2003), 18.10.5694/j.1326-5377.2003.tb05033.x12492384

74.

J. Walsh and K. Warren , ‘Selective Primary Health Care: An Interim Strategy for Disease Control in Developing Countries’, *New England Journal of Medicine*, 301, 18 (1979), 967–74; S.B. Rifkin and G. Walt, ‘Why Health Improves: Defining the Issues Concerning “Comprehensive Primary Health Care” and “Selective Primary Health Care”’, *Social Science & Medicine*, 23 (1986), 559–66.

75.

J.-P. Unger and J.R. Killingsworth , ‘Selective Primary Health Care: A Critical Review of Methods and Results’, Social Science & Medicine, 22 (1986), 1001–1013.352656410.1016/0277-9536(86)90200-5

76.

L. Gibson , ‘National Community Health Worker Programs: How Can They be Strengthened?’, Journal of Public Health Policy, 10, 4 (1989), 518.2621254

77.
‘The Community Health Worker: Report on a UNICEF/WHO Interregional Study and Workshop, 1979/1980’. PHC 80.2. WHO Library.

78.
SHS 82.3 Review of Primary Health Care Development, 99. WHO Library.

79.
SHS/HMD/84.1 ’Inter-Regional Study on Community Health Workers: A Report on the WHO Inter-Regional Study and Workshop, 4–8 July 1983, Manila Philippines’. WHO Library.

80.

Stephen Frankel (ed.), The Community Health Worker: Effective Programmes for Developing Countries (Oxford: Oxford University Press, 1992), 9.

81.

Immerwahr, *op. cit.* (note 21), 178.

82.

*Ibid*.

83.

*World Health Forum*, 5 (1984), 295.

84.

Mark Nichter , Global Health: Why Cultural Perceptions, Social Representations, and Biopolitics Matter (University of Arizona Press, 2008), 109.

85.

WHO Study Group on Community Health Workers and WHO, Strengthening the Performance of Community Health Workers in Primary Health Care: Report of a WHO Study Group [Meeting Held in Geneva from 2 to 9 December 1987](Geneva: World Health Organization, 1989), 6 http://www.who.int/iris/handle/10665/39568.2497585

86.

Eleuther Tarimo and E.G. Webster , Primary Health Care Concepts and Challenges in a Changing World (Geneva: WHO, 1997), 19–22. http://www.who.int/iris/handle/10665/62650.

87.

WHO Study Group on Community Health Workers and WHO, *op. cit.* (note 85), 10–14.

88.

*Ibid*., 6.

89.

*Ibid*., 5, reference 1.

90.

*Ibid*., 10–21.

91.
‘Wisdom and Guidance from the Community: Interview with Haile Mariam Kahssay’, *World Health*, May–June 1992, 5.

92.

World Health Organization Regional Office for the Western Pacific, WHO/UNICEF Regional Workshop on Information, Education and Communication on Health, Manila, Philippines, 17–23 March 1981: Final Report(Manila: WHO Regional Office for the Western Pacific, 1981), 123–128. http://www.who.int/iris/handle/10665/207248.

93.

World Health Organization Regional Office for the Western Pacific, Media Seminar on Health for All Through Primary Health Care, Philippines and Republic of Korea, 18–23 April 1983: Report(Manila: WHO Regional Office for the Western Pacific, 1983), 3 http://www.who.int/iris/handle/10665/207974.

94.

Consultative Group on the Organization of Health Systems Based on Primary Health Care, Report on the Meeting of the Consultative Group on the Organization of Health Systems Based on Primary Health Care, 25–29 November 1985(Geneva: World Health Organization, 1986), 5–6, 15. http://www.who.int/iris/handle/10665/59389.

95.
Letter from Dr D. Flahault, Chief Medical Officer for Health Team Development, Division of Health Manpower Development, to Dr Robert Haubrich, Consultant, Staff Development Branch, Alberta Health & Social Development, Canada, dated 18 June 1975, E 3/134/2 – Training of Health Auxiliaries – General, WHO Archives.

96.

Frankel, *op. cit.* (note 80), 7.

97.
Alma-Ata Film, Subject 3, WHO Archives.

98.
Letter to Regional Director, dated 28 April 1981, Photographic Archives. Sub-fonds Photographers, 1981, PHC-Mongolia-Henrioud, WHO Archives.

99.
Travel report summary dated 3 July 1981, Photographic Archives. Sub-fonds Photographers, 1981, PHC-Mongolia-Henrioud, WHO Archives.

100.

*World Health*, March 1990, 16–18.

101.
Letter requesting attention to the photo exhibit and video presentation in light of the technical discussions – 8 February 1994 – W3-87-10 (47). Technical Discussions for 47th WHA – 1994: Community Action for Health, WHO Archives.

102.
Minutes of 4th and 5th meeting of the Task Force for the Technical Discussion of the World Health Assembly 1994, October 1993. W3-87-10 (47), Jacket 2, WHO Archives.

103.

Tarimoand Webster, *op. cit.* (note 86), 60.

104.

Andrea Brighenti , ‘Visibility: A Category for the Social Sciences’, Current Sociology, 55 (2007), 333.

105.

C. McInnes and K. Lee , Global Health and International Relations (Cambridge: Polity Press, 2012).

106.

J. Shiffman , ‘A Social Explanation for the Rise and Fall of Global Health Issues’, *Bulletin of the World Health Organization*, 87 (2009), 608–13; S. Rushton and O.D. Williams, ‘Frames, Paradigms and Power: Global Health Policy-making Under Neoliberalism’, *Global Society*, 26 (2012), 147–67; João Nunes, ‘Ebola and the Production of Neglect in Global Health’, *Third World Quarterly*, 37, 3 (2016), 542–56.10.2471/BLT.08.060749PMC273326519705011

107.

S. Lewin and S. Munabi-Babigumira , ‘Lay Health Workers in Primary and Community Health Care for Maternal and Child Health and the Management of Infectious Diseases’, *Cochrane Database of Systematic Reviews* (2010); J.N. Brownstein, G.R. Hirsch, *et al.*, ‘Community Health Workers “101” for Primary Care Providers and Other Stakeholders in Health Care Systems’, *Journal of Ambulatory Care Management*, 34 (2011), 210–20; H. Schneider, U. Lehmann *et al.*, ‘The Global Pendulum Swing Towards Community Health Workers in Low- and Middle-income Countries: A Scoping Review of Trends, Geographical Distribution and Programmatic Orientations, 2005 to 2014’, *Human Resources for Health*, 14 (2006).

108.

James Campbell and Kesetebirhan Admasu , ‘Maximizing the Impact of Community-based Practitioners in the Quest for Universal Health Coverage’, *Bulletin of the World Health Organization*, 93, 9 (2015), 590; Prabhjot Singh and Dave Chokshi, ‘Community Health Workers: An Opportunity for Reverse Innovation – Authors’ Reply’, *The Lancet*, 382, 9901 (2013), 1327.10.1016/S0140-6736(13)62136-424139118

109.
Frontline Health Workers Coalition, *A Commitment to Community Health Workers: Improving Data for Decision Making* (2014), 5. https://www.frontlinehealthworkers.org/wp-content/uploads/2014/09/CHW-Report.pdf, accessed 22/3/2017; L. Baatiema, A.M. Sumah, *et al.*, ‘Community Health Workers in Ghana: The Need for Greater Policy Attention’, *BMJ Global Health*, 1, 4 (2016), e000141.10.1136/bmjgh-2016-000141PMC532138728588981

110.
‘Photo Contest: “Health Workers Count: A Day in the Life of a Health Worker”’, http://www.who.int/workforcealliance/forum/2013/3gf_photocontest/en/; ‘Winning photo announced’, http://resyst.lshtm.ac.uk/news-and-blogs/winning-photo-announced.

